# Autonomic effects of electroacupuncture in opioid‐induced constipation in male rats

**DOI:** 10.14814/phy2.70391

**Published:** 2025-10-14

**Authors:** Misaki Okada, Kazunori Itoh, Hiroshi Kitakoji, Kenji Imai

**Affiliations:** ^1^ Department of Acupuncture and Moxibustion Meiji University of Integrative Medicine Kyoto Japan; ^2^ Department of Acupuncture Takarazuka University of Medical and Health Care Takarazuka Japan; ^3^ Department of Acupuncture and Moxibustion, Faculty of Health Science Teikyo Heisei University Toshimaku Japan

**Keywords:** autonomic nervous system, colonic transit, electroacupuncture, loperamide, opioid‐induced constipation

## Abstract

The efficacy of electroacupuncture (EA) for opioid‐induced constipation (OIC) has been reported; however, the mechanism has not been sufficiently studied, and the details have not been clarified. The aims of this study were to demonstrate the delay in rat colonic transit (CT) caused by loperamide, a peripheral μ‐opioid receptor agonist, and to evaluate the effect of EA at ST‐36 on the autonomic nervous system. To measure CT, the operation had been demonstrated for setting the cannula into the cecum to connect the proximal colon. On the fifth day after surgery, 20 metal radiopaque markers were administered to the proximal colon and visible throughout the gastrointestinal tract via soft X‐ray immediately after the administration of markers up to 240 min. The OIC model was induced in rats by subcutaneous injection of loperamide twice a day for 3 days, and EA at ST‐36 was performed before measurement. The delay in CT caused by loperamide was significantly improved with EA, which was abolished by atropine. CT delay was significantly accelerated by α‐adrenoceptor antagonists and acetylcholinesterase inhibitors. This study showed that EA has the potential to be effective for OIC, and that the autonomic nervous system is involved in its mechanism.

## 1 INTRODUCTION

Approximately 90% of the patients with moderate to severe chronic pain are treated with opioids (Benyamin et al., 2008). Opioid receptor agonists are widely used in clinical practice because they are highly effective in the treatment of pain. However, opioids affect the physiology of the central nervous system (CNS) and peripheral nervous system (PNS), inducing constipation (Halawi et al., 2018). The combined effects of opioid receptors on gastrointestinal motility and secretion can cause opioid‐induced constipation (OIC), which occurs in 40%–95% of patients treated with opioids and can occur even with a single dose of morphine (Swegle & Logemann, 2006). Although often dismissed as a trivial side effect, the long‐term consequences of constipation can result in significant morbidity and mortality with adverse effects on a patient's quality of life. Severe constipation can force patients to reduce their opioid dose, resulting in decreased analgesia (Benyamin et al., 2008).

Acupuncture has been clinically used for constipation for several years, and electroacupuncture (EA) has shown a high efficacy rate in treating chronic functional constipation (Liu et al., 2016; Wang et al., 2020; Wang & Yin, 2015; Xu et al., 2024). Reportedly, colonic transit (CT) in normal rats is increased by EA via the parasympathetic nervous system (Iwa, Matsushima, et al., 2006). Enhancement of the parasympathetic pathway, which involves the vagus and pelvic nerves, increases colonic motility (Tong et al., 2010). EA has also been used clinically for OIC, contributing to pain management and improving the patients' quality of life by reducing constipation symptoms (Han et al., 2021; Olson et al., 2021; Wang, Liu, et al., 2023). However, the mechanism of this effect has not been sufficiently studied, and the details have not been clarified.

We established an innovative method using radiopaque markers under X‐rays to measure CT in rats over a time series in the same individual (Okada et al., 2021). Understanding colonic regulation to measure CT in a time‐course analysis is essential because it is complicated by the presence of innervation. The effects of the autonomic nervous system as an extrinsic regulator were visually observed using this method on normal rat CT, and it was reported that EA improved colonic disorders caused by autonomic nerve imbalance associated with surgery, the mechanism of which was related to an increase in the parasympathetic nerve (Okada et al., 2019). This method can measure CT over a time series to determine the therapeutic effect on colonic dysfunction and its mechanism of action.

Loperamide is used as an obstipant and acts on peripheral μ‐receptors in the enteric plexus to decrease gastrointestinal motility and digestive fluid (Awouters et al., 1983; Ho et al., 2003; Lay et al., 2016). By generating an OIC model using loperamide (Kim et al., 2019), it is possible to exclude the pain aspect and focus on physiological dysfunction. This study aimed to demonstrate the delay in CT caused by loperamide‐induced constipation using our established measurement method and to evaluate the effect of EA on the autonomic nervous system.

## 2 MATERIALS AND METHODS

### 2.1 Animals

Sixty‐four male Sprague–Dawley (SD) rats (weight, 158–303 g) were obtained from a commercial supplier (CLEA Japan, Inc., Tokyo, Japan). The animals were individually housed under conditions of controlled temperature (22–24°C), humidity, and light (12:12 h light/dark cycle, with the light cycle starting at 07:00 a.m.), with ad libitum access to laboratory chow (MF, Shimizu Laboratory Supplies Co., Ltd., Kyoto, Japan) and water. All rats were housed under standard conditions to acclimatize for at least 7 days before experimentation. All procedures were performed according to the United Kingdom Home Office Guidelines on Animals (Scientific Procedures) Act of 1986, and the protocols were approved by the Animal Research Committee at the Meiji University of Integrative Medicine, Kyoto, Japan.

### 2.2 Surgery procedure

Rats were anesthetized with 2% isoflurane. After a midline abdominal incision was made, an indwelling silastic cannula (inner diameter, 2 mm; outer diameter, 4 mm) was inserted into the cecum and positioned to enter the proximal colon. The proximal part of the tube was moved through the left abdominal wall and tunneled beneath the skin to the posterior neck, where it was fixed to the skin.

### 2.3 Measurement of colonic transit

The OIC model (Lop group) was induced in SD rats by subcutaneous injection of loperamide (4 mg/kg weight) dissolved in acetic acid with saline (0.5 mL) twice a day for 3 days, whereas the non‐constipation group (vehicle group) was injected with acetic acid with saline (0.5 mL) alone (Kim et al., 2019).

Experiment 1 (*n* = 32): To observe the effect of EA on OIC, rats were divided randomly into four groups as follows: Lop (*n* = 8), vehicle (*n* = 8), EA before measurement (Lop + EA [*n* = 8]), and EA before measurement with administration of atropine (Lop + Atropine + EA [*n* = 8]).

Experiment 2 (*n* = 32): To observe the pathophysiology of OIC via the autonomic nervous system, the rats were divided randomly into the following four groups: Lop (*n* = 8), administration of naloxone methiodide before measurement (Lop + Naloxone [*n* = 8]), administration of neostigmine before measurement (Lop + Neostigmine [*n* = 8]), and administration of phentolamine before measurement (Lop + Phentolamine [*n* = 8]).

Twenty silver metal radiopaque markers (spherical shape; diameter, 1.5 mm, SK‐301C Medical Human And A Co. Ltd., Shiga, Japan) were administered from the in‐dwelling silastic cannula to the proximal colon with 1.0 mL of saline on the fifth day after surgery. The markers were observed through the entire large intestinal tract with soft X‐ray visualization under light anesthesia with 2% isoflurane for approximately 2 min, from immediately after the administration of the markers to 240 min. Images were captured every 60 min under light anesthesia with 2% isoflurane. The animals were housed individually with ad libitum access to laboratory food and water. After the measurement of CT, 1 mL of barium (7212031B1031 FUSHIMI Pharmaceutical Co., Ltd. Kagawa, Japan) was administered to the proximal colon and was visible on imaging of the entire colon. The rats were free to move into their cages under no anesthetic influence and had ad libitum access to water between the periods of X‐ray imaging.

### 2.4 Calculation of the geometric center

**FIGURE 1:**
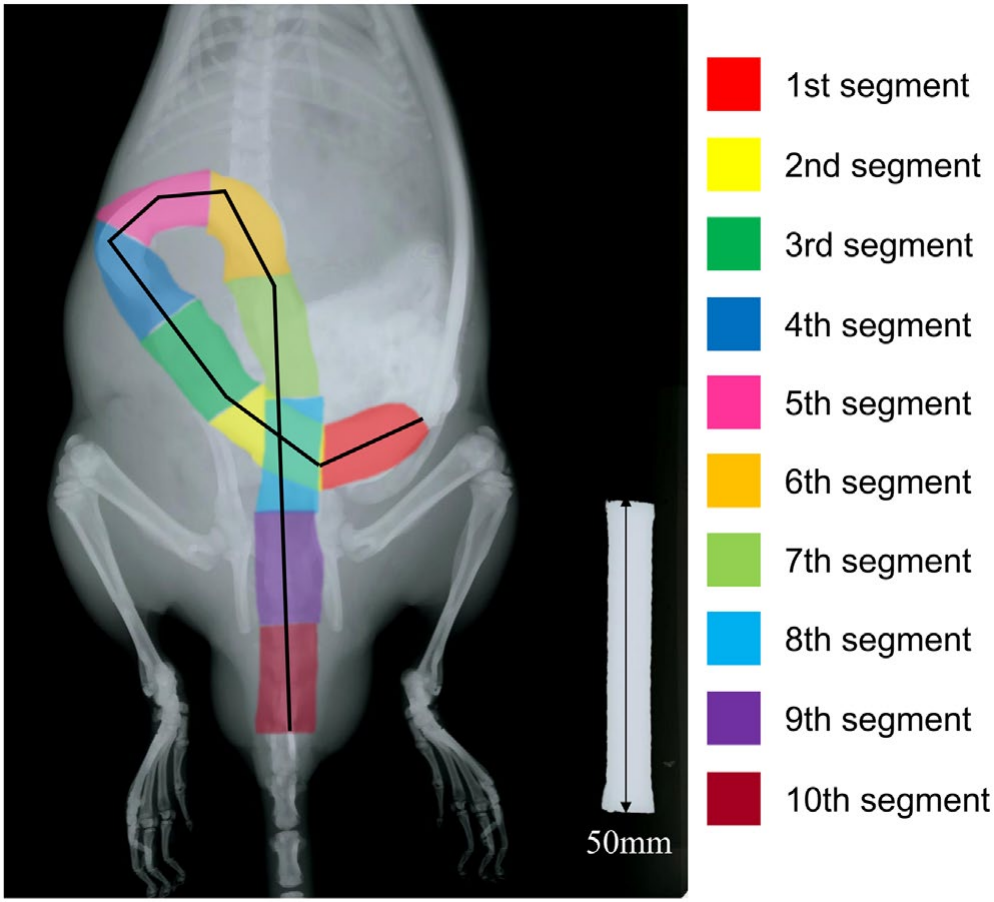
Imaging of segmentation on colon to calculate the geometric center (GC). The colon was divided into 10 equal segments. The geometric center (GC) of distribution of the radiopaque markers within the colon was calculated using the following formula for each X‐ray image: GC = Σ(number of markers) × (segment number). Scale bar = 50 mm.

After processing the images using GIMP version 2.10.3, the distance covered by the marker was calculated and the colon was divided into 10 equal segments individually (ImageJ 1.54 g, National Institutes of Health, Bethesda, MD, USA). The segmentation was only for post‐cecal portions of the colon (Figure 1). The geometric center (GC) of distribution of the radiopaque markers within the colon was calculated using the following formula for each X‐ray image: GC = Σ(number of markers) × (segment number). The GC is expressed in arbitrary units. The investigators that did the segmentation procedure and calculations were not blinded to the animal's treatment assignment.

### 2.5 EA protocol

Hook‐shaped needles were used to avoid the spontaneous removal of the inserted acupuncture needles from the rat bodies (Imai et al., 2008; Iwa, Matsushima, et al., 2006). Before the administration of markers, stainless acupuncture needles (0.34 mm in diameter; length: 30 mm; Asahi Co., Saitama, Japan) were inserted into the skin and underlying muscles at either ST‐36 at a depth of 5 mm. ST‐36 is located approximately 5 mm lateral to and below the anterior tubercle of the tibia. The inserted needles were connected to an electrical pulse generator (Ohm Pulser LFP‐2000e, Zeniryoki Co., Fukuoka, Japan) and stimulated by an electrical current (alternating current at a frequency of 10 Hz, 0.5 ms duration, and 0.01 mA intensity) for 20 min under anesthesia maintained by isoflurane (1%).

### 2.6 Pharmacological treatment

The OIC model was generated by the subcutaneous injection of loperamide (4 mg/kg, 129–05721 Wako Pure Chemical Industries, Osaka, Japan) dissolved in acetic acid with saline (0.5 mL) twice daily for 3 days. To investigate whether the cholinergic pathway was involved in mediating the effects of EA on loperamide‐induced constipation, atropine (0.05 mg/kg, 1242405A1062 Mitsubishi Tanabe Pharma Corporation, Osaka, Japan) with saline (0.5 mL) was administered intraperitoneally immediately before starting EA. Moreover, to investigate the pathophysiology of loperamide‐induced constipation, naloxone methiodide (5 mg/kg, N129 Sigma‐Aldrich, St Louis, MO, USA), neostigmine (0.1 mg/kg, 1233400A2024 Sionogi and Co., LTD, Osaka, Japan), and phentolamine (1 mg/kg, P7547 Sigma‐Aldrich, St Louis, MO, USA) in saline (0.5 mL) were administered intraperitoneally immediately before the administration of markers.

### 2.7 Statistical analysis

The data were expressed as mean ± standard deviation (SD). All eight groups of rats were weighed before surgery and CT measurements and compared by paired *t*‐test. The Dunnet test was implemented for comparisons among multiple groups. Dunnett's test was performed to determine changes in GC in each group compared with the Lop group in Experimentation 1 and 2. A significance level of *p* < 0.05 was considered statistically significant. Data were analyzed using JMP version 12.2.0 (SAS Institute, Japan).

## 3 RESULTS

### 3.1 Changes in rat body weight before surgery and measurement

No significant weight loss was observed in all eight groups (Table 1). The Lop and vehicle groups in experiment 1 increased significantly more weight before CT measurement than before surgery.

**TABLE 1:** Changes of body weight in rats.

Experimentation 1	Lop		Vehicle		Lop + EA		Lop + Atropine+EA	
	Mean ± SD	*p* Value	Mean ± SD	*p* Value	Mean ± SD	*p* Value	Mean ± SD	*p* Value
Before surgery	240.4 ± 31.1	0.020	220.0 ± 23.1	0.015	241.6 ± 35.4	0.763	218.4 ± 31.7	0.052
Before measurement	250.3 ± 30.3		229.1 ± 28.3		240.3 ± 40.8		224.5 ± 31.2	

Experimentation 2	Lop		Lop + Naloxone		Lop + Neostigmine		Lop + Phentolamine	
	Mean ± SD	*p* Value	Mean ± SD	*p* Value	Mean ± SD	*p* Value	Mean ± SD	*p* Value
Before surgery	211.4 ± 31.2	0.088	182.3 ± 17.3	0.780	223.5 ± 31.2	0.862	213.0 ± 31.3	0.589
Before measurement	218.4 ± 28.3		184.8 ± 23.4		223.3 ± 32.7		215.5 ± 30.1	

*Note*: All eight groups of rats on experiment 1 and 2 were weighed before surgery and colonic transit (CT) measurements and compared by paired *t*‐test. *N* = 8 in each group.

### 3.2 Effects of electroacupuncture on loperamide‐induced colonic transit delay

**FIGURE 2:**
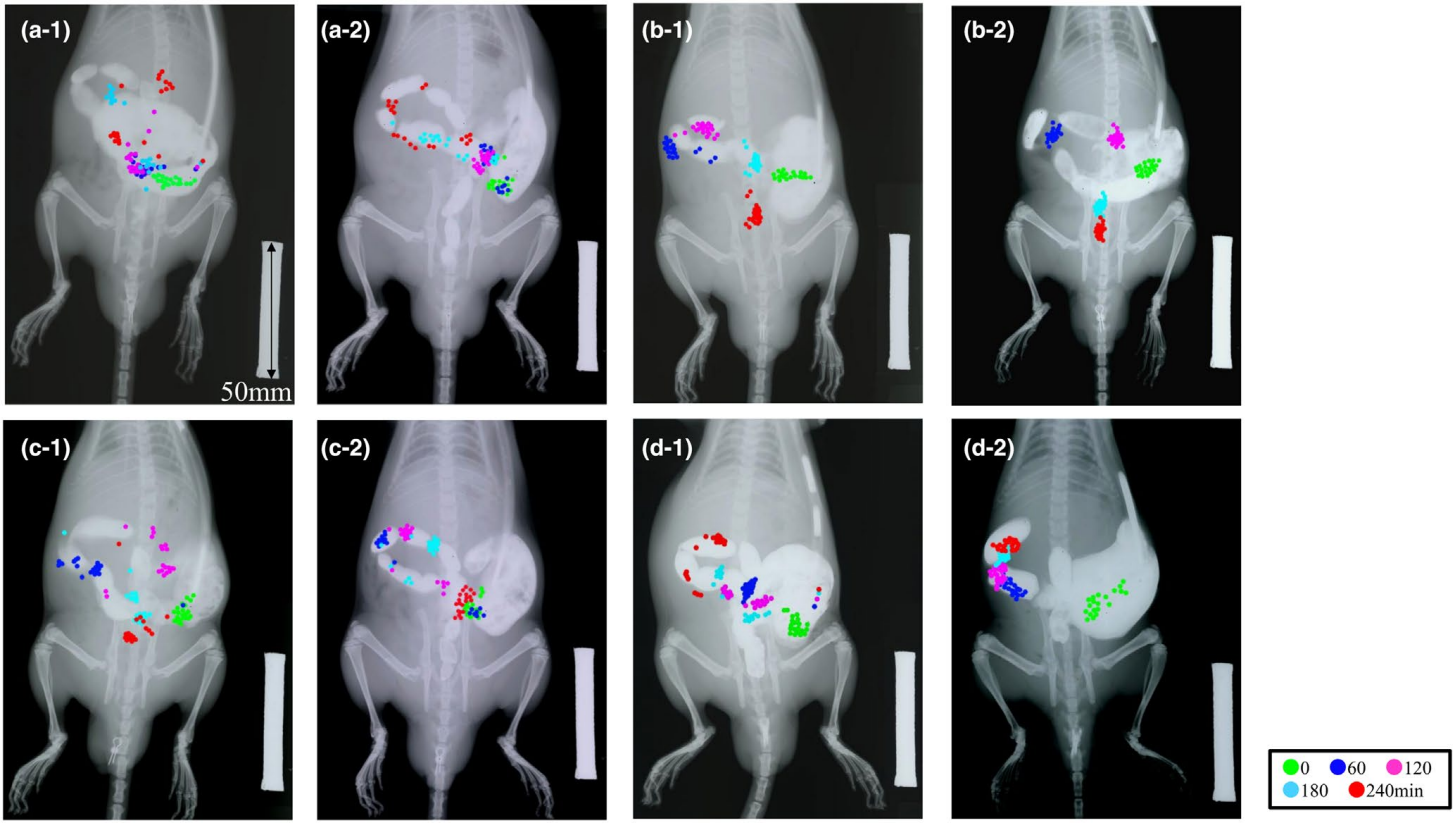
Imaging of colonic transit (CT) of radiopaque markers in each group in experiment 1. Scale bar = 50 mm. The markers are colored differently at each time point. (a‐1 and 2) Loperamide (Lop) group. (b‐1 and 2) Vehicle group. (c‐1 and 2) Lop + Electroacupuncture (EA) group. (d‐1 and 2) Lop + Atropine + EA group.

**FIGURE 3:**
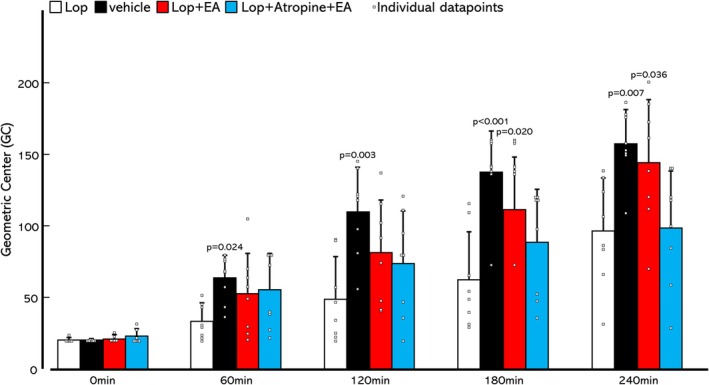
Geometric Center (GC) of each group in Experiment 1. Data are expressed as mean ± standard deviation of the mean. Dunnett's test was performed to determine changes in GC in each group compared with the Loperamide (Lop) group. *N* = 8 in each group.

Representative images of CT for all procedures in two rats from each group are shown in Figure 2, and the GC of each group is shown in Figure 3. In the Lop group (Figure 2a–1,2), most markers remained in the proximal colon from immediately after injection to 240 min. The marker moved smoothly toward the distal colon at 180 min in the vehicle group (Figure 2b–1,2). There was a significant difference in GC between the Lop and vehicle groups from 60 to 240 min after marker injection, with delayed CT in the Lop group (Figure 3, Lope and vehicle; 60 min 33.6 ± 12.1 and 63.6 ± 15.9, 120 min 48.8 ± 28.7 and 110.1 ± 30.1, 180 min 62.3 ± 33.5 and 138.4 ± 28.2, 240 min 96.8 ± 36.6 and 157.5 ± 24.1). Loperamide was shown to delay CT. The Lop + EA (Figure 2c–1,2) and Lop + Atropine + EA (Figure 2d–1,2) groups showed similar marker transitions to the vehicle and Lop groups, respectively. The GC of the Lop + EA group showed a significant difference from the Lop group at 180 and 240 min, indicating that EA improved loperamide‐induced delay of CT (Lop + EA; 180 min 111.5 ± 37.2, 240 min 144.8 ± 43.1). However, the Lop + Atropine + EA group was not significantly different from the Lop group at any time point, and the improvement in CT with EA was antagonized by atropine (Figure 3).

### 3.3 Pathogenesis of loperamide‐induced colonic transit delay

**FIGURE 4:**
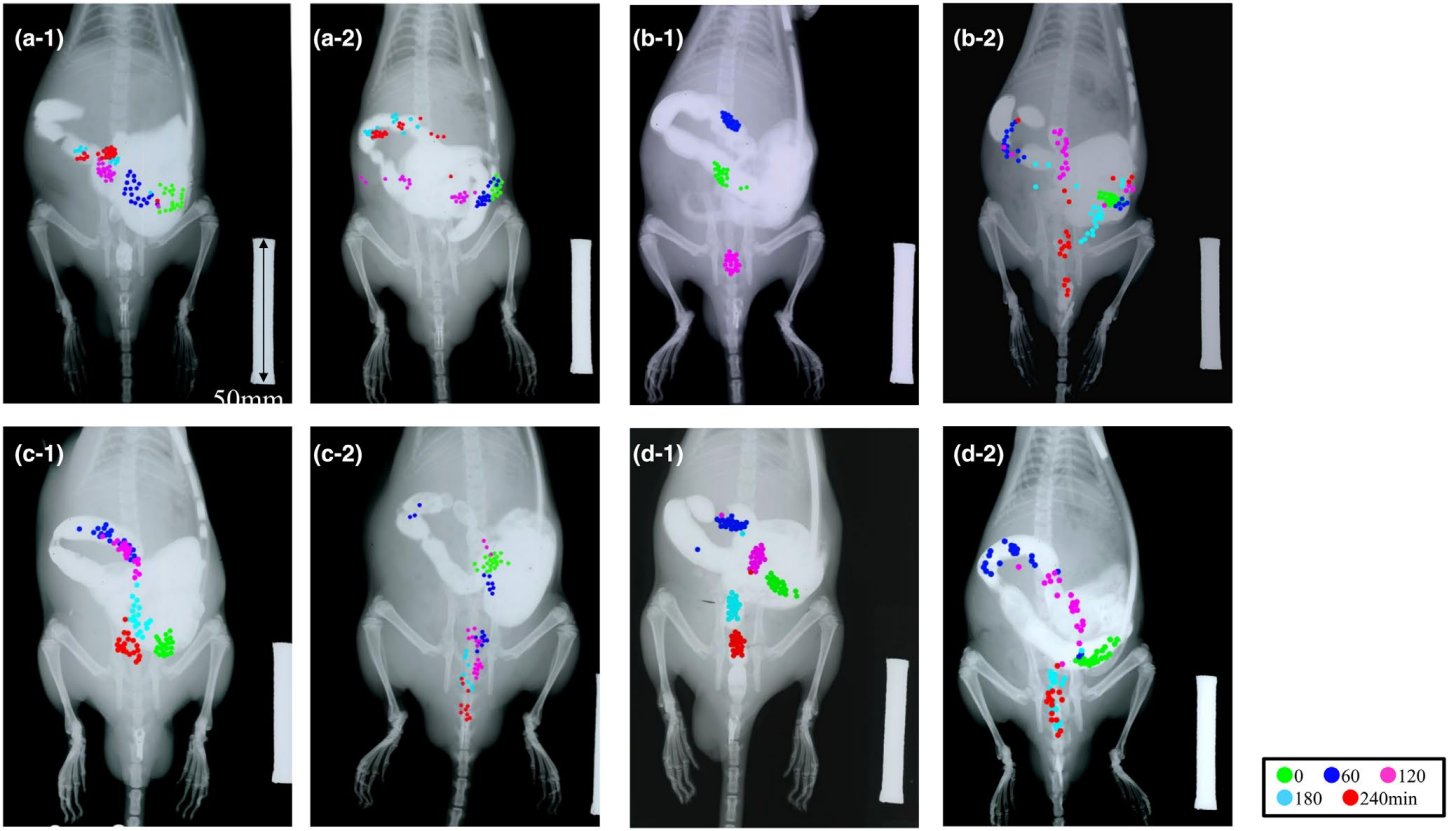
Imaging of the colonic transit (CT) of radiopaque markers in each group in experiment 2. Scale bar = 50 mm. The markers are colored differently at each time point. (a‐1 and 2) Loperamide (Lop) group. (b‐1 and 2) Lop + Naloxone group. (c‐1 and 2) Lop + Neostigmine group. (d‐1 and 2) Lop + Phentolamine group.

**FIGURE 5:**
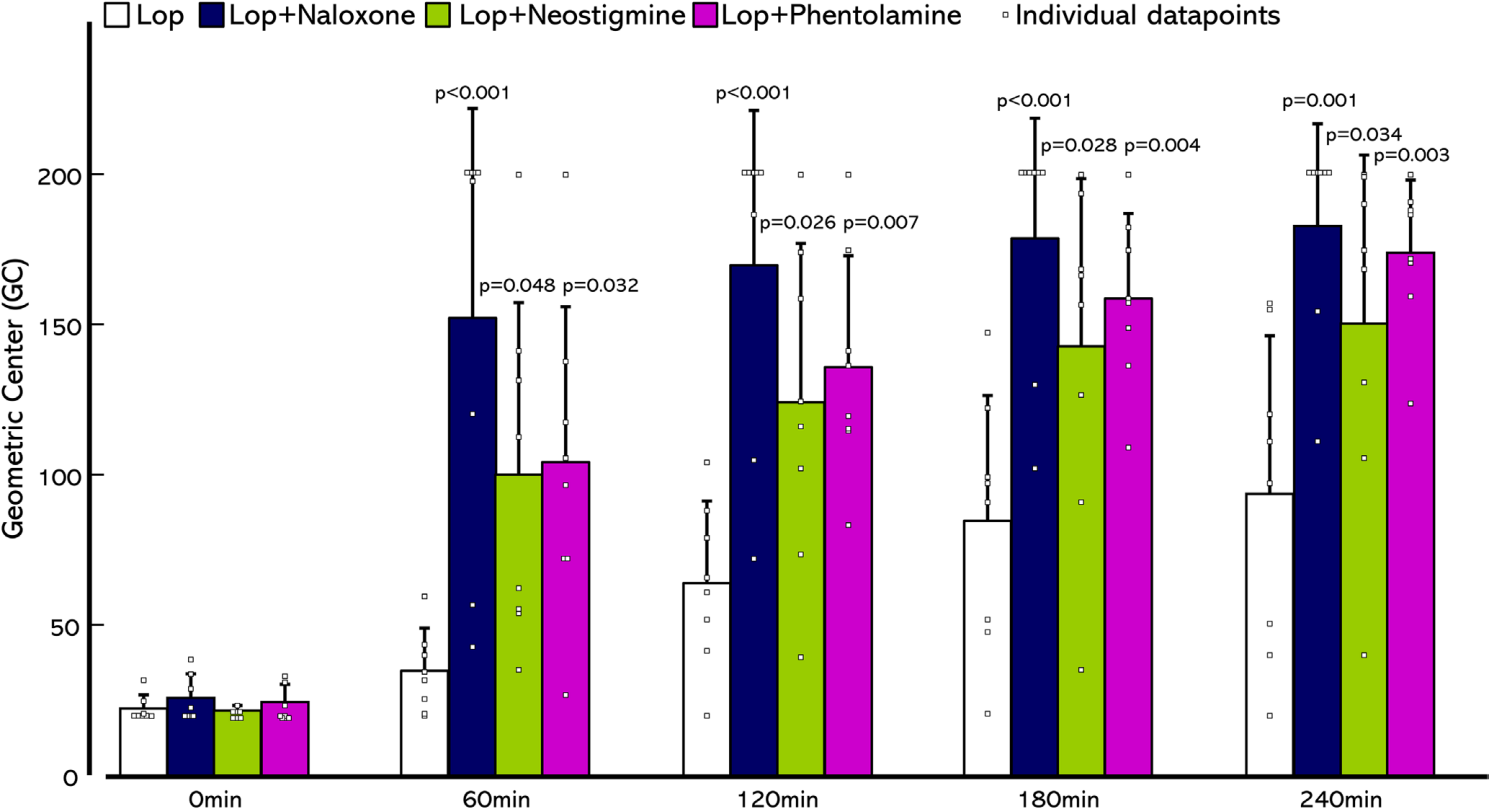
Geometric Center (GC) of each group in Experiment 2. Data are expressed as mean ± standard deviation of the mean. Dunnett's test was performed to determine changes in GC in each group compared with the Loperamide (Lop) group. *N* = 8 in each group.

Representative images of CT for all procedures in two rats from each group are shown in Figure 4, and the GC of each group is shown in Figure 5. In the Lop group (Figure 4a–1,2), most markers remained in the proximal colon from immediately after injection to 240 min and showed similar marker transitions to experiment 1. The Lop + Naloxone (Figure 4b–1,2), Lop + Neostigmine (Figure 4c–1,2), and Lop + Phentolamine groups (Figure 4d–1,2) had accelerated markers. In particular, all or some markers, which at 180 and 240 min on the Lop + Naloxone group (Figure 4b–1), at 180 and 240 min on the Lop + Neostigmine group (Figure 4c–2) and at 240 min on the Lop + Phentolamine group (Figure 4d–2), were not visible because they were ejected from the anus. All three groups, Lop + Naloxone, Lop + Neostigmine, and Lop + Phentolamine, had significantly increased GC at 60 to 240 min compared to the Lop group (Figure 5, Lop, Lop + Naloxone, Lop + Neostigmine and Lop + Phentolamine; 60 min 34.8 ± 13.3, 152.1 ± 68.8, 99.6 ± 56.5, and 104.1 ± 51.2, 120 min 64.0 ± 26.7, 170.4 ± 51.5, 124.0 ± 52.8, and 136.1 ± 36.8, 180 min 84.6 ± 41.6, 179.0 ± 39.6, 142.8 ± 55.6, and 158.9 ± 28.0, 240 min 93.9 ± 52.0, 183.1 ± 33.3, 151.4 ± 55.6 and 174.1 ± 24.0). Naloxone, Neostigmine, and Phentolamine accelerated loperamide‐induced delay of CT, respectively.

## 4 DISCUSSION

In this study, we measured CT over time in a rat model of OIC and examined the effects of EA. The results showed that CT delayed by loperamide (peripheral μ‐receptor agonist) was improved by EA and was abolished by atropine, a parasympathetic nerve blocker. Loperamide‐delayed CT was significantly enhanced by naloxone (μ‐receptor antagonist), neostigmine (parasympatholytic agent), and phentolamine (α‐receptor blocker), suggesting that EA affects the autonomic nervous system.

In this study, the surgery of silastic cannula placement required for the CT measurement was performed 5 days before the measurement. During the last 3 days of the recovery period, subcutaneous injections of loperamide were administered to create the OIC model. However, no significant weight loss was observed in each group from before surgery to before measurement. Moreover, the Lop and vehicle group in experimentation 1 showed significant weight gain. Although the effects of surgical stress are reflected in CT on the day after surgery (Okada et al., 2019), the recovery period was considered sufficient in this study.

Opioid drugs interact with three classes of opioid receptors: μ, δ, and κ‐opioid receptors, although opioid drugs mostly target the μ‐receptors (Galligan & Sternini, 2017). The μ‐receptors are present in the enteric plexus and expressed on enteric nerves and Cajal cells in a wide area of the rat gastrointestinal tract (Bagnol et al., 1997). It has also been reported to be present in both the submucosal and myenteric nerve plexuses of the small and large intestines in humans (Sternini et al., 2004). The myenteric nerve plexus is located between the circular and longitudinal muscle layers of the gut wall and includes an enteric nervous system (ENS) network of excitatory and inhibitory motor neurons and descending and ascending interneurons (Spencer et al., 2021). Intestinal myoneurons release acetylcholine and substance P to cause muscle contraction (Brookes, 2001), and the opioid receptor is linked to the inhibition of acetylcholine release from enteric interneurons and motor neurons, and purine/nitric oxide release from inhibitory motor neurons (Galligan & Sternini, 2017), causing decreased secretion of digestive juices and peristaltic activity, retarded transport of intestinal contents, and tensed anal sphincter (Camilleri et al., 2014). The side effects of opioid analgesics can lead to OIC.

In the ileum of guinea pigs, loperamide has been shown to act via μ‐opioid receptors to inhibit the excitatory motor neurons of the ENS (Waterman et al., 1992). Moreover, loperamide inhibits the release of acetylcholine, the primary excitatory neurotransmitter released by myenteric neurons, to induce muscle contractions by interacting with opioid receptor sites in the myenteric plexus (Yagasaki et al., 1978). Unlike opioids, loperamide is readily available, has a similar structure, and is often used to create OIC models (Wang, Gharibani, et al., 2023; Zhang et al., 2021; Zhao et al., 2024). This study also used loperamide to induce OIC in rats and observed the effects of EA.

We established the method to measure CT and/or time series experiments in the same individual and observed changes in CT using pharmacological techniques (Okada et al., 2021). CT is accelerated by inducing an enhanced cholinergic pathway and by significantly blocking α‐adrenoceptors. Furthermore, blocking muscarinic receptors at high concentrations delays CT. However, there were no changes in CT with β‐adrenoceptor antagonists. This study was designed to measure CT in the same manner as this method. Electrical stimulation of the cervical vagus and pelvic nerves in normal rats causes marked contractions in the mid and distal colons, which are antagonized by atropine (Tong et al., 2010). EA of ST36 enhances the movements of the rat distal colon and is antagonized by atropine (Iwa, Matsushima, et al., 2006). EA can change CT by affecting exogenous parasympathetic activity.

EA of the sacral region has already been proven effective against morphine‐induced CT delay, but its autonomic involvement is unknown (Wang, Gharibani, et al., 2023; Zhang et al., 2016). EA has been shown to be effective for gastrointestinal symptoms caused by autonomic nervous system abnormalities (Iwa, Nakade, et al., 2006; Jin et al., 2017; Li et al., 2022; Okada et al., 2019; Song et al., 2013). EA via chronically implanted electrodes in ST36 improves loperamide‐induced gastric emptying and whole‐gut transit time delay (Wang et al., 2019). In this study, the autonomic function of heart rate variability was assessed and EA demonstrated parasympathetic hyperactivity and sympathetic inhibition. The present study showed that the improvement of EA to ST36 in loperamide‐induced CT delay was achieved pharmacologically using atropine via the parasympathetic nervous system. Electrical stimulation of the auricular vagus and sacral nerves has been reported to improve CT in a rat OIC model via the parasympathetic nerves (Wang, Gharibani, et al., 2023; Zhang et al., 2021). Auricular vagal stimulation increases parasympathetic activity via the nucleus tractus solitarius (NTS) and dorsal motor nucleus of the vagus (DMV). EA of ST36 in normal rats has been reported to increase c‐fos expression in the NTS and the medial portion of the DMV, suggesting that EA of ST36 affects the CT by increasing pelvic nerve activity via the Burlington nucleus rather than the vagus nerve (Iwa, Matsushima, et al., 2006). Sacral nerve stimulation increased excitatory innervation and decreased inhibitory innervation in the loperamide‐altered ENS. One week after auricular vagal and sacral nerve stimulation, glial cell line‐derived neurotrophic factor expression increased, which is essential for ENS formation, and improved ENS function. Although 3‐d administration of loperamide also alters the tissue (Kim et al., 2019), it is unclear whether a single EA, rather than continuous EA, altered the composition of the ENS in this case. In clinical practice, patients with OIC defecate immediately after a single acupuncture treatment, suggesting that acupuncture stimulation affects the autonomic nervous system rather than structural changes in the ENS (Olson et al., 2021).

In this study, the pathogenesis of loperamide‐induced delay in CT was investigated pharmacologically. The effect of loperamide on colonic motility has been reported to be reversed by naloxone, which was similar in the present study (Parkar et al., 2024). Neostigmine, an acetylcholinesterase inhibitor, decreases colonic compliance, suggesting increased colonic tone in patients with chronic constipation who do not have megacolon (Mouchli et al., 2016). The pharmacological effect of cholinesterase inhibition by neostigmine indicates the neuromuscular responsiveness of the colon to endogenous acetylcholine, and cholinergic innervation could have improved the CT delay by loperamide with EA. Moreover, phentolamine, a nonselectiveα‐adrenoceptor antagonist, also abolished loperamide‐induced CT delay, suggesting that increased sympathetic activity via α‐adrenoceptors may promote the inhibition of acetylcholine release and smooth muscle relaxation (Stebbing et al., 2001). Suppression of sympathetic nerve activity via α‐adrenergic receptors by EA may also have contributed to the improvement in CT latency induced by loperamide.

This study had several limitations, including the use of only male rodents to prevent the confounding effects of female hormones since sex differences in colonic motility have been reported (Horii et al., 2023). Furthermore, it would have been advantageous to determine the GC measures in animals given loperamide + atropine together, in order to discern the extent of additional slowing vs. loperamide alone. Additionally, only CT was assessed in this study; therefore, stool water content and stool frequency, as well as gastric and small bowel motility, were unknown. Nevertheless, this study showed that EA has the potential to be effective for OIC and that the autonomic nervous system is involved in its mechanism. The results of this study could contribute to the treatment of adverse side effects in patients treated with opioids.

## AUTHOR CONTRIBUTIONS

O.M. and I.K. conceived and designed research; O.M. performed experiments; O.M. and Imai.K. analyzed data; Itoh. K. and K.H. interpreted results of experiments; O.M. prepared figures; O.M. drafted manuscript; I.K. edited and revised manuscript; all authors approved final version of manuscript.

## FUNDING INFORMATION

This work was supported by JSPS KAKENHI Grant Number 20K16562.

## CONFLICT OF INTEREST STATEMENT

No conflicts of interest, financial, or otherwise, are declared by the authors.

## ETHICS STATEMENT

All procedures were approved by the Animal Research Committee at the Meiji University of Integrative Medicine (2021‐002, 2022‐003, 2022‐008 and 2023‐005).

## Data Availability

The data of this study are available upon reasonable request from the corresponding author.
